# γ-Aminobutyric Acid (GABA) Metabolic Bypass Plays a Crucial Role in Stress Tolerance and Biofilm Formation in *C. sakazakii* ATCC 29544

**DOI:** 10.3390/foods14020171

**Published:** 2025-01-08

**Authors:** Jiangchao Wu, Yigang Yu, Fengsong Liu, Yifang Cao, Jiahao Ren, Yiting Fan, Xinglong Xiao

**Affiliations:** 1 The College of Life and Geographic Sciences, Kashi University, Kashi 844000, China; wujiangchao2008@163.com (J.W.); yuyigang@scut.edu.cn (Y.Y.); 2 School of Food Science and Engineering, South China University of Technology, Guangzhou 510640, China; 202320126445@mail.scut.edu.cn (J.R.); 202410186963@mail.scut.edu.cn (Y.F.); 3 Guangxi Key Laboratory of Clean Pulp & Papermaking and Pollution Control, School of Light Industrial and Food Engineering, Guangxi University, Nanning 530004, China; liufengsong@gxu.edu.cn

**Keywords:** *Cronobacter sakazakii*, GABA, *gabT*, environmental stress, biofilm

## Abstract

*Cronobacter sakazakii* is a foodborne pathogen characterized by its robust stress tolerance and ability to form biofilms, which facilitates its survival in powdered infant formula (PIF) processing environments for prolonged periods. Gamma-aminobutyric acid (GABA) is a kind of non-protein amino acid that acts as an osmoprotectant. This study aimed to elucidate the effects of the *gabT* gene on the survival of *C. sakazakii*, GABA accumulation, and biofilm formation under desiccation, osmotic stress, and acid exposure. A *gabT* knockout strain of *C. sakazakii* was developed using gene recombination techniques. The GABA content and survival rates of both the wild-type and knockout strains were compared under various stress conditions. Scanning electron microscopy (SEM) was used to observe cellular damage and biofilm formation. Statistical analysis was performed using a one-way analysis of variance (ANOVA). The deletion of *gabT* resulted in enhanced GABA accumulation under different stress conditions, improving the bacterium’s tolerance to desiccation, osmotic pressure, and acid treatment. SEM images revealed that under identical stress conditions, the *gabT* knockout strain exhibited less cellular damage compared to the wild-type strain. Both strains were capable of biofilm formation under low osmotic pressure stress, but the *gabT* knockout strain showed higher GABA content, denser biofilm formation, and increased biofilm quantity. Similar trends were observed under acid stress conditions. The *gabT* gene plays a key role in modulating GABA accumulation, which enhances the stress tolerance and biofilm formation of *C. sakazakii*. These findings provide new insights into the role of GABA in bacterial survival mechanisms and highlight the potential for targeting GABA pathways to control *C. sakazakii* in food processing environments.

## 1. Introduction

*Cronobacter sakazakii* is a Gram-negative pathogenic bacterium belonging to the *Enterobacteriaceae* family, posing a significant life-threatening risk to specific populations. *C. sakazakii* is associated with meningitis, bacteremia, and small intestinal colitis, with mortality rates ranging from 40% to 80% [1]. Although patients infected with *C. sakazakii* can recover following antibiotic treatment, they often experience neurological sequelae and developmental disorders [2]. Studies have identified contaminated powdered infant formula (PIF) as a major contributor to infections in infants and young children [3]. Furthermore, it has been suggested that the long-term survival of *C. sakazakii* in PIF can be attributed to two main factors, its strong resistance to adverse conditions and its ability to form biofilms [4].

*C. sakazakii* demonstrates a remarkable ability to survive under adverse environmental conditions, including desiccation, osmotic pressure, and acidity. It exhibits superior resistance to desiccation stress compared to other members of the *Enterobacteriaceae* family, which enhances its long-term survival in low-water-activity foods, such as PIF [5]. Research shows that while pathogens such as *Salmonella*, *Escherichia coli*, and *Klebsiella pneumoniae* can survive in PIF for up to 15 months, *C. sakazakii* can persist for over 2.5 years [6]. Furthermore, this pathogen exhibits significant resistance to osmotic stress. A study investigating the salt stress tolerance of 15 strains of *C. sakazakii* revealed heterogeneity in survival across different strains in media containing 4–10% NaCl, with clinical isolates demonstrating strong tolerance to osmotic pressure [7]. Further research confirmed that certain strains of *C. sakazakii* are capable of growth in environments containing 10% NaCl [8]. As for acid tolerance, the pH of adult gastric fluid typically ranges from 1.5 to 3.5; however, neonatal gastric fluid can have a pH as high as 5.17. This higher pH increases the likelihood of survival for pathogens, including *C. sakazakii* [9]. It has been reported that *C. sakazakii* is tolerant to acidic conditions, with tolerance levels varying depending on the strain, acid strength, and species involved.

*C. sakazakii* has been documented to form biofilms on abiotic surfaces, including food processing equipment [10,11] and apparatus involved in the reconstitution of powdered infant formula [12]. An increased synthesis of extracellular polysaccharides during biofilm formation provides physical defense for bacterial cells, enabling them to withstand the stressful conditions encountered during food processing [13]. This characteristic enhances the bacteria’s survival and dissemination, posing a significant challenge for prevention and control in the food industry. Therefore, investigating the resistance of *C. sakazakii* to adversity and its ability to form biofilms is crucial for developing effective strategies to inactivate this bacterium during food processing.

**Figure 1:**
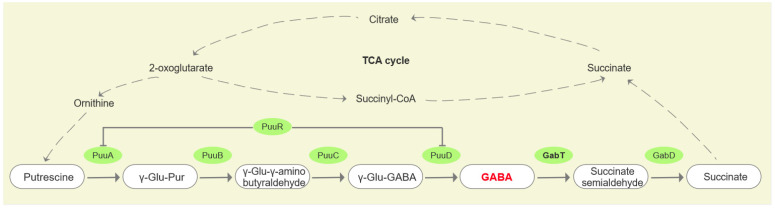
Schematic representation of the role of the *gabT* gene in the GABA metabolic bypass, GABA is highlighted in bold red font.

γ-aminobutyric acid (GABA) is a non-protein amino acid commonly found in microorganisms, animals, and plants. The γ-aminobutyric acid transaminase gene (*gabT*) plays a critical role in the GABA metabolic bypass (Figure 1). In higher plants, multiple studies have shown that the GABA shunt is an important mechanism for drought tolerance, with stress conditions often leading to significant GABA accumulation [14]. For instance, Bao et al. [15] demonstrated that silencing the *gabT* gene in tomato resulted in elevated GABA levels, reduced succinic acid content, and enhanced osmotic tolerance. It has been revealed that a flagellin-induced GABA shunt improves drought stress tolerance in *Brassica napus* L [16]. In addition, the GABA shunt is crucial in microbial responses to environmental stresses, such as acid tolerance [17], general stress response [18], and pathogenic bacterial virulence [19]. Additionally, mutations in *gabT* have been associated with reduced pathogenicity in *Pseudomonas syringae* [20].

Proteomic technology using iTRAQ has been conducted to investigate desiccation stress tolerance in *C. sakazakii* ATCC 29544, and the findings revealed a significant upregulation of enzymes related to putrescine metabolism, specifically PuuR, PuuA, and PuuD, indicating an increase in GABA synthesis under desiccation stress [21]. This suggests that the accumulation of GABA derived from the degradation of putrescine and other polyamines could be a vital mechanism for desiccation tolerance in *C. sakazakii*. Although the role of GABA in desiccation tolerance is well established, most research has focused on plants rather than bacteria; its specific function in *C. sakazakii* remains unclear. In particular, it is not well understood whether the *gabT* gene can influence GABA production through metabolic regulation in this bacterium nor how such regulation might affect its stress resistance and biofilm formation abilities.

In this study, a *gabT* gene knockout strain of *C. sakazakii* ATCC 29544 was constructed using gene knockout technology. The survival rates, biofilm formation, and intracellular GABA content of both the WT strain and the knockout strain were analyzed under various stress conditions. These experiments aimed to validate the critical role of the GABA metabolic shunt in stress resistance and biofilm formation in *C. sakazakii*. This study provides novel evidence regarding the role of GABA in osmotic and acid stress in *C. sakazakii*, an area in which experimental data are limited in the existing literature. We hypothesize that disrupting the GABA metabolic shunt significantly impairs the ability of *C. sakazakii* to withstand environmental stress and form biofilms. The findings provide a foundation for developing targeted regulatory strategies based on the GABA shunt, offering new insights for the inactivation of *C. sakazakii* in the food industry.

## 2. Materials and Methods

### 2.1. Construction of Gene Knockout Mutants

The *gabT* knockout strain of *C. sakazakii* was prepared following a previously established method [22]. Primers were designed based on the sequences of *gabT* and the plasmid, with details provided in Table S1. The upstream and downstream fragments of the *gabT* gene were obtained through amplification, after which the *gabT* fusion fragment was generated via overlapping PCR. The plasmid was constructed and transformed into E. coli β2163 using the recombinant enzyme Exnase II to ligate the products of the suicide vector pLP12cm and the fusion fragment. Finally, the *gabT* mutant was screened through two times of homologous recombination, and the deletion mutant was cloned and submitted for the sequencing of the PCR product.

### 2.2. Culture Conditions

*C. sakazakii* ATCC 29544 and Δ*gabT* were stored in tryptic soy broth (TSB) with 30% glycerol at −80 °C. The activation of the strain was achieved by inoculating the culture medium on TSA and cultured at 37 °C for 24 h. Then, a single colony of each strain was transferred into 100 mL TSB and the mixture was incubated at 180 rpm 37 °C for 18 h.

### 2.3. Growth Curves Assay

Growth curves were measured to verify the effect of gene knockout on the growth of the *C. sakazakii* strain. After activation, either WT or Δ*gabT* strains were inoculated into TSB by transferring a single colony from TSA plates using an inoculating loop. The cultures were then incubated at 37 °C in a shaker set to 180 rpm. Optical density at 600 nm (OD_600_) was measured every two hours using a microplate reader (Thermo Fisher, Shanghai, China).

### 2.4. Stress Tolerance Assay

For the preparation of bacterial suspension, cultures in the logarithmic growth phase were collected and centrifuged at 8000 rpm for 5 min to remove the supernatant. The bacterial pellet was then washed three times with PBS and various concentrations of bacterial suspensions were prepared using a 10-fold serial dilution method. The reconstitution of PIF (RIF) was prepared according to the manufacturer’s instructions.

#### 2.4.1. Desiccation Stress

Ten grams of PIF and 1 mL of bacterial suspension were added to a sterile bag. The mixture was kneaded for 2 min to ensure thorough blending of the bacterial solution with the milk powder. Subsequently, the sample was homogenized using a homogenizing beater to achieve a final contamination level of approximately 6 log_10_ CFU/g for each strain. After homogenization, the sample was placed in a desiccator containing potassium acetate for equilibration [23]. The sample remained in the desiccator for 5 days to reach a water activity level close to 0.25, during which viable counts were assessed.

#### 2.4.2. Osmotic Stress

The culture of each strain was separately suspended and diluted with RIF containing 9% NaCl (*w/v*) to obtain an initial population of 6 log_10_ CFU/mL. Then, the culture was incubated at room temperature (25 °C) for 0, 1, 2, 3, 4, 5, and 6 h [22]. After incubation, those samples were centrifuged and diluted with 0.9% (*w/v*) saline solution to achieve proper concentrations.

#### 2.4.3. Acid Stress

To simulate the acidic environment of the stomach, simulated gastric fluid (SGF, pH = 3.4) was used to obtain acid cells of *C. sakazakii*. SGF was prepared as described by Wu [24]. Cells of different strains were subjected in SGF solution to obtain an initial flora of about 6 log_10_ CFU/mL. Then, the cultures were incubated in a shaker (150 rpm) at 37 °C for different incubation times (0, 1, 2, 3, 4, and 5 h).

After drying, osmotic pressure, or acid stress treatment, the samples were washed three times with PBS, diluted to the appropriate concentration, and then spread onto TSA plates for viable bacteria counting. The survival rate was calculated using the following formula:

Survival rate (%) = (total number of viable bacteria after stress treatment/total number of viable bacteria before stress treatment) × 100%.

### 2.5. Determination of GABA Content

The GABA content in *C. sakazakii* WT and Δ*gabT* strains was measured according to the instructions provided with the GABA assay kit (Merck Biotech, Shanghai, China). The preparation of stress-treated samples for each strain followed the method described in Section 2.4. This kit employs an enzyme-linked immunosorbent assay (ELISA) to quantify GABA content, involving the formation of an antibody–antigen–enzyme complex between the GABA antibody, GABA, and the horseradish peroxidase (HRP)-labeled GABA antibody. After thorough washing, the substrate tetramethylbenzidine (TMB) was added, which is catalyzed by HRP to produce a color change from blue to yellow upon acid addition. The color intensity is directly proportional to the GABA concentration. The optical density at 450 nm (OD_450_) was measured using a microplate reader, and GABA concentration in each sample was calculated based on the standard curve.

### 2.6. Biofilm Formation Assay

Biofilm formation was assessed using the crystal violet staining method as previously described [25]. The bacterial suspensions of the WT and Δ*gabT* strains were prepared according to Section 2.2, with the turbidity of the suspensions adjusted to a McFarland standard of 0.5 using sterile physiological saline (0.9% NaCl, *w/v*). Subsequently, 20 μL of bacterial suspensions were inoculated into 96-well plates made of polystyrene containing 180 μL of TSB medium and incubated at 37 °C for 48 and 72 h. The wells were then gently washed three times with phosphate-buffered saline (PBS) and stained with 0.1% crystal violet for 30 min in the dark. After washing with PBS three times, the stain was solubilized with 95% ethanol, and the optical density at 595 nm (OD_595_) of the supernatant in each well was measured using a microplate reader (Bioteck, Synergy Neo2, Arcugnano, Italy). Each sample was tested in triplicate, and the average value was calculated. TSB without bacteria served as the negative control.

### 2.7. Determination of Biofilm Formation by SEM

Following the method of Liu et al. [26], scanning electron microscopy (SEM, Hitachi, S-3400N-II, Tokyo, Japan) was used to examine the effects of acid and osmotic stress on the biofilm morphology of *C. sakazakii*. WT and Δ*gabT* strains were inoculated onto six-well plates with sterilized coverslips, and the samples were incubated at 37 °C for 48 h. Biofilms formed by WT and Δ*gabT* strains were fixed onto the coverslips with 2.5% glutaraldehyde for at least 2.5 h. The coverslips were then washed three times with sterile PBS and sequentially dehydrated with ethanol at increasing concentrations (10%, 30%, 50%, 70%, 90%, and 100%). The coverslips were then attached to the SEM-specific specimen holders using specialized conductive adhesive, followed by dehydration and gold sputtering. After metallization, the samples were observed under a Hitachi S-3400N-Ⅱscanning electron microscope to examine the microscopic structural morphology of the biofilms. The magnification used for observing the biofilms was 2500×.

### 2.8. Statistical Analysis

All experiments were conducted in triplicate with independent biological replicates. Standard deviations are indicated in the plots by error bars. Statistical comparisons were performed using a one-way ANOVA for independent samples in SPSS version 26. Tukey’s post hoc test was used to determine significant differences between groups (*p* < 0.05).

## 3. Results

### 3.1. Growth Curve

**Figure 2:**
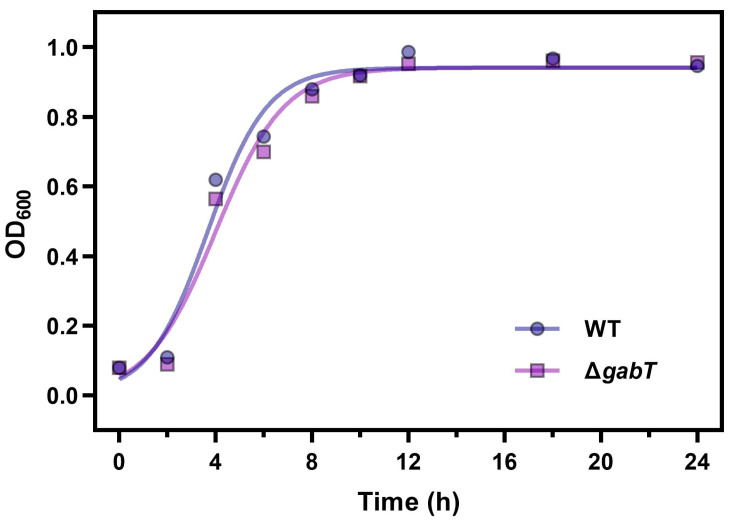
Growth curves of WT and Δ*gabT* under normal conditions.

The growth dynamics of *C. sakazakii* ATCC 29544 WT and Δ*gabT* strains in TSB were analyzed using growth curves. As shown in Figure 2, there was no significant difference in the overall growth between WT and Δ*gabT* strains. The Δ*gabT* strain exhibited normal growth and proliferation similar to the WT strain under optimal conditions, with both strains entering the logarithmic and stationary phases simultaneously. This finding suggests that the deletion of the *gabT* gene does not significantly impact the growth of *C. sakazakii* in a suitable environment and the metabolic pathways regulated by *gabT* are not critical for bacterial growth under these conditions.

### 3.2. Survivability and GABA Content Under Desiccation Stress Conditions

**Figure 3:**
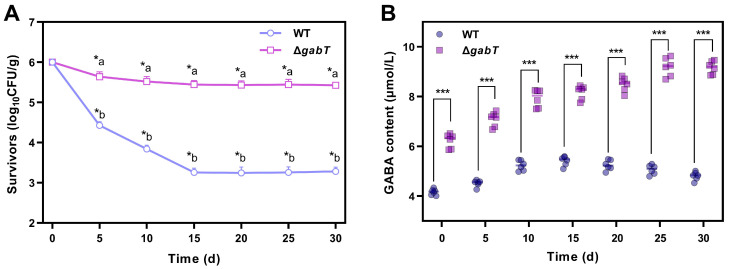
Survivability (**A**) and changes in GABA content (**B**) of WT and Δ*gabT* under desiccation stress conditions. ^a,b^ Mean values differ significantly between two strains in the same desiccation conditions (*p* < 0.05); * mean values differ significantly in different desiccation conditions of each strain (*p* < 0.05); *** mean values differ significantly between two strains in the same desiccation conditions (*p* < 0.05).

As depicted in Figure 3A, both WT and Δ*gabT* strains exhibited a significant decline in survival rate following desiccation treatment. After ten days of drying, the survivors of the WT strain decreased from 6 to 3.84 log_10_ CFU/g, while the viable counts of the Δ*gabT* strain decreased to 5.52 log_10_ CFU/g. After twenty days of drying, the survival rate of the WT strain further declined to 3.24 log_10_ CFU/g, whereas the survivability of the Δ*gabT* strain decreased to 5.43 log_10_ CFU/g. Following this period, the survivors of both strains stabilized, with the WT and Δ*gabT* strains exhibiting a survivability of 3.25 and 5.44 log_10_ CFU/g, respectively. These findings suggest that the desiccation stress tolerance of *C. sakazakii* is enhanced upon the knockdown of *gabT*.

The changing trends in GABA content in *C. sakazakii* WT and Δ*gabT* strains is illustrated in Figure 3B. Under desiccation stress, GABA levels increased in both strains. After fifteen days of drying, the GABA content in Δ*gabT* rose from 6.23 μmol/L to 8.17 μmol/L, whereas the WT strain exhibited an increase from 4.17 μmol/L to 5.42 μmol/L. By thirty days, the Δ*gabT* strain achieved a GABA content of 9.17 μmol/L, while the GABA content in the WT strain remained stable between the fifteen- and thirty-day marks, reaching approximately 5 μmol/L at the end. These findings indicate that the Δ*gabT* strain maintained a higher GABA content under drying stress compared to the WT strain.

### 3.3. Survivability and GABA Content Under Osmotic Stress Conditions

**Figure 4:**
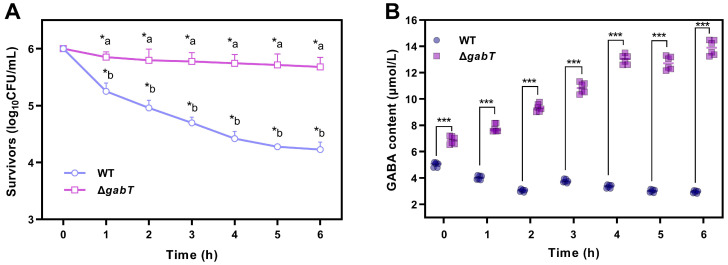
Survivability (**A**) and changes in GABA content (**B**) of WT and Δ*gabT* under osmotic stress conditions. ^a,b^ Mean values differ significantly between two strains in the same osmotic conditions (*p* < 0.05); * mean values differ significantly in different osmotic conditions of each strain (*p* < 0.05); *** mean values differ significantly between two strains in the same osmotic conditions (*p* < 0.05).

The osmotic stress tolerance of WT and Δ*gabT* strains in RIF medium demonstrated significant declines in viable bacterial counts (*p* < 0.05) across treatments (Figure 4A). After 3 h of osmotic stress treatment, the survival count of the WT strain decreased to 4.70 log_10_ CFU/mL, whereas the Δ*gabT* strain exhibited minimal change, decreasing from 6.00 to 5.78 log_10_ CFU/mL. Similarly, after 6 h of incubation in RIF, the survival rates of the Δ*gabT* and WT strains dropped to 5.68 and 4.23 log_10_ CFU/mL, respectively. The WT strain exhibited a significantly lower survival rate compared to the Δ*gabT* strain (*p* < 0.05).

Under 9% NaCl osmotic stress, the GABA content in the Δ*gabT* strain increased significantly over time, while the GABA content in the WT strain decreased. After 6 h of treatment, the GABA content in the Δ*gabT* strain rose from 6.87 to 13.89 μmol/L, nearly doubling, whereas the GABA content in the WT strain declined from 5.08 μmol/L to 2.62 μmol/L (Figure 4B).

### 3.4. Survivability and GABA Content Under Acid Stress Conditions

**Figure 5:**
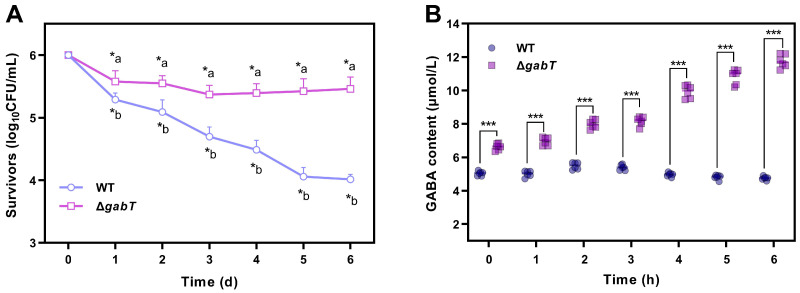
Survivability (**A**) and changes in GABA content (**B**) of WT and Δ*gabT* under acid stress conditions. ^a,b^ Mean values differ significantly between two strains in the same acid conditions (*p* < 0.05); * mean values differ significantly in different acid conditions of each strain (*p* < 0.05); *** mean values differ significantly between two strains in the same acid conditions (*p* < 0.05).

In this study, the survival of WT and Δ*gabT* strains under the acidic stress of simulated gastric fluid at pH 3.4 decreased during the first 3 h (Figure 5A). Both strains experienced a significant decline in survival following acid stress treatment (*p* < 0.05). Notably, the viable count of Δ*gabT* strains began to increase after 3 h of acid exposure. After 6 h of treatment, the survivability of Δ*gabT* decreased to 5.46 log_10_ CFU/mL, while that of WT dropped to 4.01 log_10_ CFU/mL. Overall, the survival rate of Δ*gabT* under acid stress was significantly higher than that of WT (*p* < 0.05).

Additionally, the changes in GABA content during the growth of WT and Δ*gabT* under acid stress conditions (pH 3.4) were evaluated (Figure 5B). As the duration of acid stress treatment increased, the GABA content in the WT strain exhibited a decreasing trend, whereas the GABA content in the Δ*gabT* strain gradually increased. After 6 h of acid stress treatment, the GABA content in the WT strain was 4.74 μmol/L, while in the Δ*gabT* strain, it reached 11.75 μmol/L, which was significantly higher than that of the WT strain (*p* < 0.05). The increase in GABA content was consistent with the observed survival trends of the two strains under acid stress conditions.

### 3.5. Effect of Stress Treatment on Bacterial Cell Morphology

**Figure 6:**
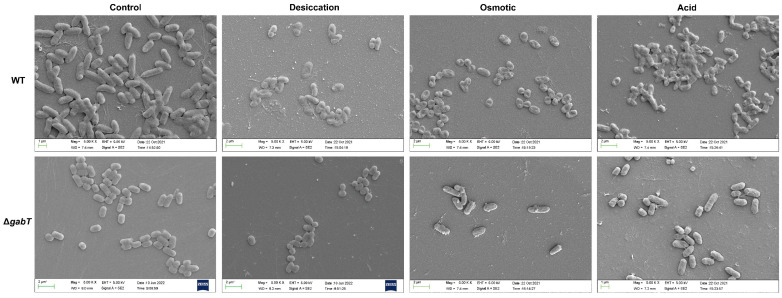
Morphological changes observed by field emission scanning electron microscopy of different samples under normal, desiccation, osmotic, and acid stress conditions.

Under normal growth conditions, both the WT and Δ*gabT* strains exhibited intact and robust cell morphology (Figure 6). However, under desiccation stress, the WT strain displayed noticeable surface holes, while the Δ*gabT* strain showed signs of cell shriveling without significant damage. In osmotic stress conditions, the WT strain exhibited pronounced cell shrinkage and damage, whereas only slight damage was observed on both sides of the Δ*gabT* cells. Under acid stress, the WT strain experienced severe cell damage, characterized by a visible leakage of intracellular contents, while the Δ*gabT* strain underwent shrinkage with holes observed on both sides. These observations suggest that the Δ*gabT* strain is more capable of maintaining cell morphology under stress conditions, which may contribute to its higher survival rates, consistent with the results from the survival experiments.

### 3.6. Effects of Osmotic Stress on Biofilm Formation and GABA Content

**Figure 7:**
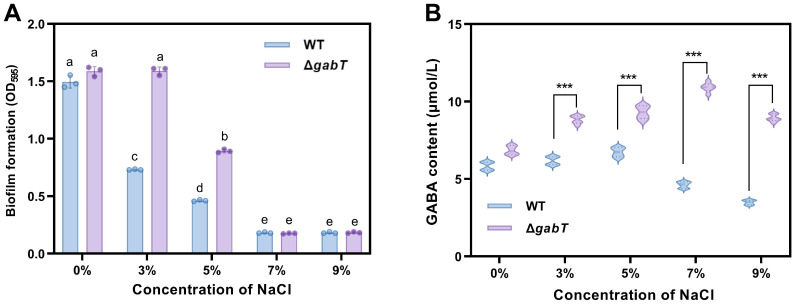
Biofilm formation (**A**) and changes in GABA content (**B**) of WT and Δ*gabT* under normal or osmotic stress conditions. ^a–e^ Mean values differ significantly among different samples; *** mean values differ significantly between two strains in the same osmotic conditions.

In this study, crystal violet staining was employed to quantify the formation of biofilms by *C. sakazakii*. Under normal growth conditions, the Δ*gabT* strain demonstrated a slightly enhanced capacity for biofilm formation compared to the WT strain (*p* > 0.05, Figure 7A). In the low-salt-concentration treatment group (3% NaCl), the biofilm formation of the Δ*gabT* strain remained largely unchanged compared to the control samples. Conversely, the biofilm formation of the WT strain under this osmotic stress condition (OD_595_ = 0.73) was significantly lower than that of the control group (OD_595_ = 1.49). When the NaCl concentration was increased to 5%, biofilm formation decreased in both strains; however, the Δ*gabT* strain (OD_595_ = 0.89) exhibited significantly greater biofilm formation than the WT strain (OD_595_ = 0.46). As the NaCl concentration was further increased to 9%, the biofilm formation for both strains decreased to approximately 0.18.

As illustrated in Figure 7B, under identical osmotic pressure conditions, the GABA content in the Δ*gabT* strain consistently surpassed that of the WT strain, with the most significant difference observed at a salt concentration of 7%. At a NaCl concentration of 5%, the GABA content in the WT strain began to decline, while the GABA content in the Δ*gabT* strain exhibited a reduction only at salt concentrations exceeding 7%.

### 3.7. Effects of Osmotic Stress on the Biofilm Microstructure

**Figure 8:**
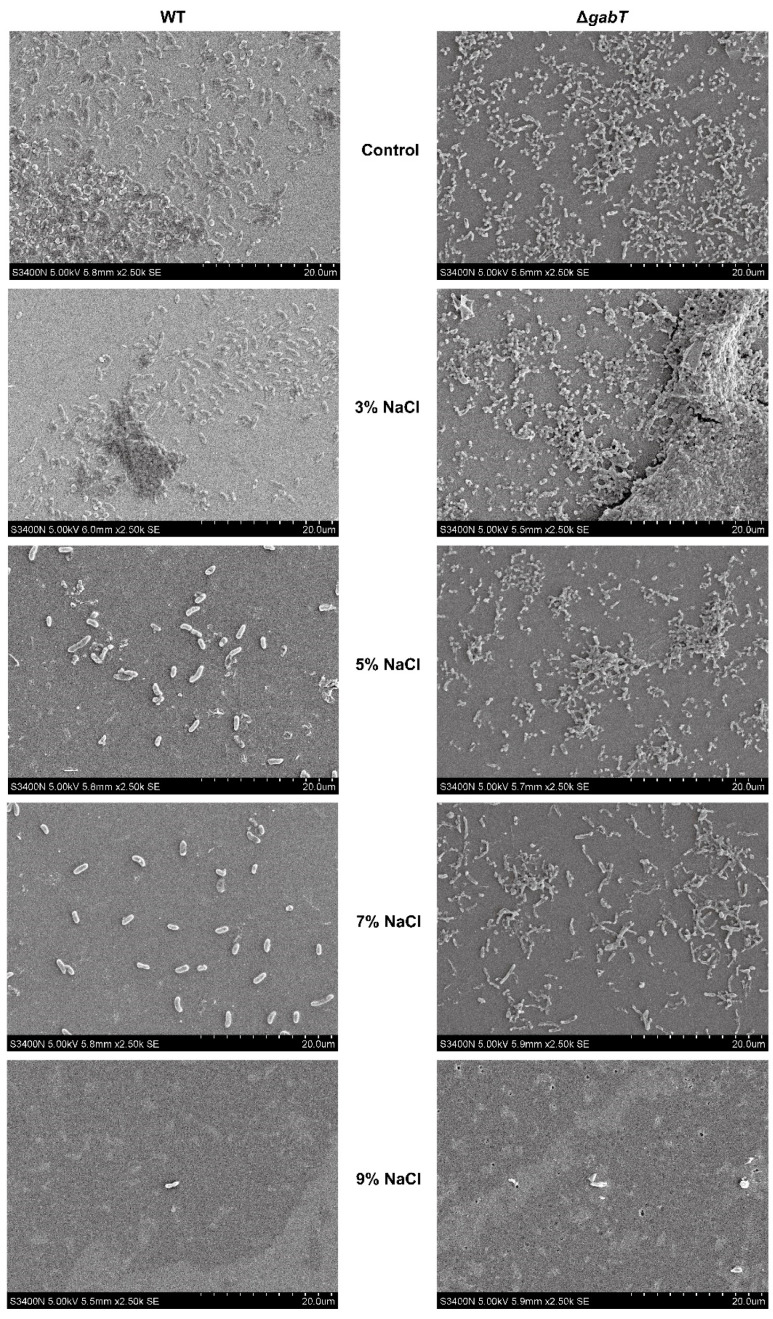
Biofilms formed on glasses of WT and Δ*gabT* under normal or osmotic stress conditions by SEM.

The SEM images in Figure 8 reveal the changes in biofilms formed by *C. sakazakii* under osmotic stress. After 48 h of incubation, both the Δ*gabT* and WT strains aggregated into multilayer stacks, demonstrating a distinct three-dimensional structure. Following treatment with 3% NaCl, the Δ*gabT* strain exhibited a denser biofilm and an increase in extracellular polymer production, which aligns with the findings from the crystal violet assay. As osmotic pressure increased (NaCl concentrations above 5%), biofilm formation decreased for both *C. sakazakii* strains, resulting in bacterial cells appearing as a sparse monolayer. Notably, the Δ*gabT* strain maintained higher biofilm formation and greater extracellular polymer content compared to the WT strain. At a NaCl concentration of 9%, fragments of broken cells were observed within the field of view.

### 3.8. Effects of Acid Stress on Biofilm Formation and GABA Content

**Figure 9:**
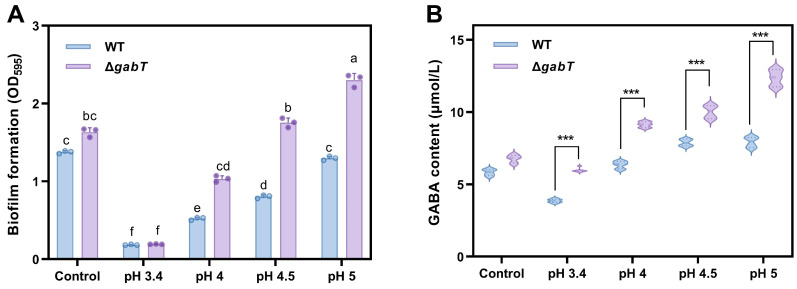
Biofilm formation (**A**) and changes in GABA content (**B**) of WT and Δ*gabT* under normal or acid stress conditions. ^a–f^ Mean values differ significantly among different samples; *** mean values differ significantly between two strains in the same acid conditions.

With increasing acidity in the stress environment, the biofilm formation ability of *C. sakazakii* WT gradually declines. For the Δ*gabT* strain, both biofilm formation and extracellular polymer content under acidic conditions (pH 3.4 and pH 4) were significantly lower than those in untreated samples (*p* < 0.05). However, as the pH rose to 5.0, biofilm formation by Δ*gabT* exceeded that of the control group, reaching an OD_595_ value of 2.30 compared to 1.63 in the control. These results suggest that a mildly acidic environment can promote biofilm formation in the Δ*gabT* strain (Figure 9A).

As illustrated in Figure 9B, GABA content increased in both the WT and Δ*gabT* strains under acid stress conditions. At a pH of 5, the GABA content in WT and Δ*gabT* strains was measured at 7.92 and 12.37 μmol/L, respectively, with the Δ*gabT* strain demonstrating a significantly higher GABA concentration than the WT strain (*p* < 0.05). As the pH decreased, the accumulation of GABA progressively diminished in comparison to the control group. At a pH of 3.4, the GABA content in the WT and Δ*gabT* strains was recorded at 3.86 and 5.98 μmol/L, respectively, both of which were lower than the GABA content observed in the control conditions.

### 3.9. Effects of Acid Stress on the Biofilm Microstructure

**Figure 10:**
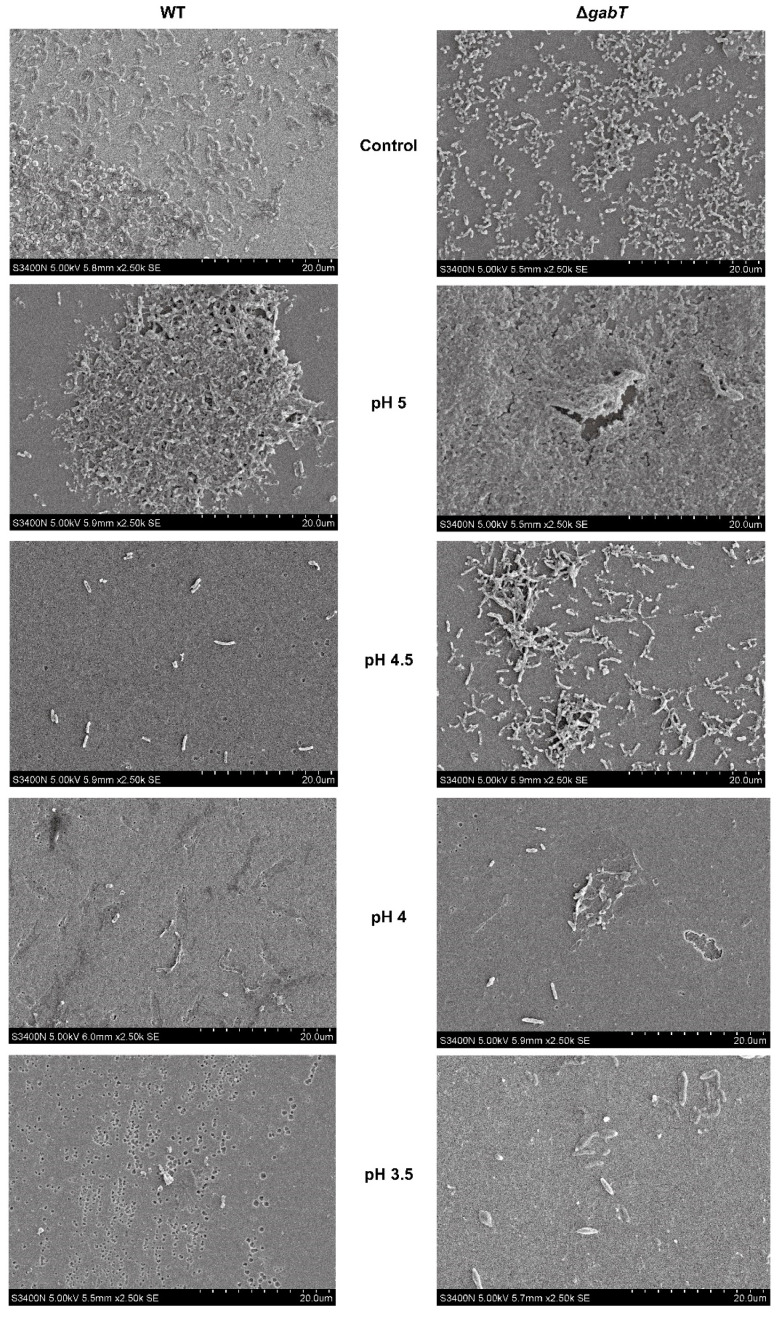
Biofilms formed on glasses of WT and Δ*gabT* under normal or acid stress conditions by SEM.

Under acid stress, changes in the biofilm morphology of *C. sakazakii* are evident in SEM images (Figure 10). At pH 3.4, damaged bacteria and lysed cells are visible but no distinct biofilm structure is present. As the pH increases, biofilms appear denser, with richer extracellular polymers. At pH 4.5, notable bacterial aggregation is observed, while at pH 5, extensive biofilm formation and a dense layer of extracellular polymers dominate the field of view. Across all acidic conditions, Δ*gabT* consistently showed a greater biofilm formation ability than WT, consistent with the crystal violet staining results.

## 4. Discussion

*C. sakazakii* is a common opportunistic pathogen found in PIF and exhibits resistance to various environmental pressures. Consequently, a deeper understanding of the potential tolerance mechanisms of *C. sakazakii* could aid in its activation. This study hypothesized that the GABA metabolic shunt plays a critical role in the stress resistance and biofilm formation of *C. sakazakii*. Based on the experimental results, this hypothesis was accepted. Specifically, the survival rates, GABA accumulation, and biofilm formation ability of the *gabT* knockout strain were significantly higher under osmotic, desiccation, and acid stress conditions compared to the WT. These findings provide strong evidence supporting the hypothesis. Specific discussions of these results are described below.

### 4.1. GABA Accumulation Improve the Ability of Desiccation Tolerance

In this study, desiccation stress significantly increased GABA levels in both Δ*gabT* and WT strains, with the Δ*gabT* strain exhibiting a markedly higher accumulation than WT (Figure 3). After ten days of drying, the GABA content in the Δ*gabT* strain rose from 6.23 μmol/L to 7.93 μmol/L (*p* < 0.05), while in the WT strain, it increased from 4.17 μmol/L to 5.23 μmol/L (*p* < 0.05). Desiccation is typically associated with high osmotic stress, and *C. sakazakii* responds through a phased regulatory mechanism. Initially, cells increase intracellular osmotic pressure by accumulating electrolytes such as potassium ions and glutamate to counteract external osmotic forces. However, prolonged high ion concentrations may lead to ionotoxicity, so a secondary response involves synthesizing or absorbing compatible solutes, like proline and glycine betaine, which stably remain in cells, mitigate ion toxicity, and provide osmotic balance [27,28]. As a small-molecule osmotic regulator, GABA increases cytoplasmic osmotic potential, thereby enhancing cell water retention and reducing desiccation-induced damage [29].

Further analysis revealed that after twenty days of drying, the GABA level in the Δ*gabT* strain rose to 8.49 μmol/L, while the GABA level in the WT strain remained relatively stable, reaching approximately 5 μmol/L at the end (*p* < 0.05). This discrepancy suggests that the deletion of the *gabT* gene in *C. sakazakii* impairs GABA transaminase activity, hindering further GABA metabolism and facilitating its intracellular accumulation. The elevated GABA accumulation in Δ*gabT* under desiccation stress aligns with its improved desiccation resistance. Studies indicate that strains with high desiccation tolerance exhibit an increased expression of genes related to the synthesis and transport of compatible solutes (e.g., bet*AB*, *proVW*) compared to low-tolerance strains, suggesting that such gene expression enhances desiccation resilience [28]. Thus, the substantial GABA accumulation observed in Δ*gabT* under desiccation in this study not only reflects the effects of the genetic mutation but also enhances the survival rate of Δ*gabT* in dry conditions. This evidence underscores that GABA accumulation may play a critical protective role under desiccation stress, conferring the Δ*gabT* strain with significantly greater resistance than WT.

### 4.2. GABA Accumulation Improves the Ability of Osmotic Tolerance

Osmotic pressure is commonly utilized in the food processing industry as a method to inhibit pathogenic bacteria. The concentration process in PIF production involves osmotic pressure stress. Existing research on the mechanisms by which bacterial cells resist osmotic stress often draws analogies between dry and hypertonic environments [30]. When exposed to hypertonic conditions, *C. sakazakii* relies on the accumulation of intracellular electrolytes to elevate osmotic pressure and counterbalance the external hypertonic environment [31].

GABA, a four-carbon non-protein amino acid, exists as a zwitterion under normal physiological pH conditions and is readily soluble in water. Its biochemical properties resemble those of small osmotic molecules such as proline and betaine. Consequently, GABA serves as an osmotic adjustment substance that increases osmotic water potential in the cytoplasm, enhances cellular water retention, and mitigates damage caused by dehydration [14]. In this study, the Δ*gabT* strain exhibited a higher accumulation of GABA under osmotic stress (Figure 4B), correlating with its improved survival rate (Figure 4A). The deletion of the *gabT* gene in Δ*gabT* disrupts GABA metabolism, leading to its accumulation, which aids the cells in resisting osmotic stress and results in a higher survival rate compared to WT. Desiccation strategies of *C. sakazakii* in low-moisture environments have been summarized; the accumulation of osmoprotectants or the production of chaperone proteins that prevent oxidative damages are the main strategies [1]. Therefore, in the Δ*gabT* strain, GABA accumulation enhances osmotic water potential, ultimately increasing survival rates. The higher survival rate of *C. sakazakii* in PIF may be attributed to the protective effects of lipids, proteins, and other components present in PIF.

### 4.3. GABA Accumulation Improves the Ability of Acid Tolerance

Gastric acid serves as the first line of defense against bacterial infections in the host. The survival rate of *C. sakazakii* during the rehydration of PIF and in simulated gastric fluid was assessed, and the evidence confirms that *C. sakazakii* can grow under acidic conditions [32,33]. Currently, while the relationship between GABA content and stress tolerance in plants has been extensively studied, research on the role of *gabT* in bacterial environmental tolerance remains limited. Few studies have reported that GABA enhances the acid resistance of *Listeria monocytogenes* [17]. In the present study, both the WT and Δ*gabT* strains exhibited decreased survivors within the first three hours under acid stress of pH 3.4 (Figure 5A); however, the number of Δ*gabT* strains began to increase after this period. Furthermore, the *gabT* mutant demonstrated higher viability than the WT throughout the acid tolerance conditions. The glutamate decarboxylase (GAD) system is primarily associated with resistance to acidic conditions in microorganisms. This system produces GABA as a by-product, which serves as a substrate in the GABA shunt pathway [34]. The GAD system contributes to intracellular pH balance by consuming protons during the decarboxylation reaction [35], utilizing glutamate to produce GABA. Consequently, microorganisms exploit this mechanism to remove protons from the intracellular environment under acidic conditions. The authors of [17] reported that GABA accumulation is crucial for the survival of *Listeria monocytogenes* under acidic stress, with increased GABA levels correlating with enhanced survival at low pH values, a finding consistent with the results of this study. In the Δ*gabT* strain, the disruption of the downstream metabolic pathway for GABA prevented succinate from being dehydrogenated to produce hydrogen ions, further aiding *C. sakazakii* in responding to acid stress.

### 4.4. GABA Accumulation Improves the Ability of Biofilm Formation

*C. sakazakii* is known to develop biofilms rapidly as a self-protective response to adverse environmental conditions [5]. Research has demonstrated that extracellular polysaccharides can protect cells within biofilms from environmental stressors [36]. In this study, under acid (pH 5) or osmotic conditions (3% NaCl), *C. sakazakii* exhibited higher extracellular polymer content and a denser tissue structure compared to the untreated group (Figure 8 and Figure 10). The biofilm matrix encapsulating *C. sakazakii* consists of extracellular polysaccharides, proteins, and extracellular DNA (eDNA), which collectively create mechanical stability and cohesion among the bacteria [21]. This stability enhances the survival and persistence of microorganisms within biofilms [5]. As illustrated in Figure 7A and Figure 9A, *C. sakazakii* resists environmental stress by forming a biofilm under conditions of weak acid and hypo-osmolality. However, as the pH decreases, the harsh environment negatively impacts *C. sakazakii* growth, resulting in decreased survival rates and subsequently reduced extracellular polymer production. Notably, regardless of treatment conditions, Δ*gabT* exhibited greater extracellular polymer content than WT, correlating with its higher survival rate under acid stress. The Δ*gabT* strain demonstrated not only enhanced survival but also greater viable bacterial counts and increased extracellular polymer secretion, which provide better protection against acidic environments. Similarly, under low osmotic pressure, this strain can resist stress through biofilm formation. However, as osmotic pressure increases, viable bacterial numbers and activity decline, preventing effective biofilm formation, which aligns with survival rate results. Furthermore, measurements of GABA content in the planktonic cells of the biofilm revealed that the deletion of the *gabT* gene disrupts GABA metabolism, leading to GABA accumulation (Figure 7B and Figure 9B). This accumulation aids in environmental stress resistance, resulting in denser biofilm formation in the Δ*gabT* strain compared to the WT strain.

In summary, this study confirms for the first time that the GABA metabolic shunt contributes to desiccation, osmotic, and acid stress resistance in *C. sakazakii*. These findings not only offer new insights into the stress adaptation mechanisms of this pathogen but also establish a foundation for developing targeted strategies to prevent and control *C. sakazakii* contamination in food processing. Despite these contributions, certain limitations of the study should be acknowledged. For instance, the research focused on a single strain of *C. sakazakii*, and the broader applicability of these findings across other strains or environmental conditions remains to be explored. Additionally, while this study highlights the role of GABA in stress resistance and biofilm formation, the underlying biochemical mechanisms, particularly at the molecular level, require further investigation. Future studies should aim to elucidate the GABA-mediated regulatory pathways and their interactions with other metabolic processes in *C. sakazakii*.

## 5. Conclusions

In this study, the *gabT* mutant of *C. sakazakii* was constructed using homologous recombination techniques to investigate its role in environmental stress tolerance and biofilm formation. Comparative analyses of survival rates, biofilm formation, and GABA content between the wild-type strain and the *gabT* mutant revealed that GABA enhances *C. sakazakii*’s tolerance to desiccation, osmotic pressure, and acidity while promoting biofilm formation under stress conditions. These findings confirm that *gabT* plays a critical role in the environmental tolerance and biofilm development of *C. sakazakii*. This study provides a foundation for developing strategies to reduce the stress resistance and biofilm formation of *C. sakazakii* in food processing environments. Future research should focus on elucidating the molecular mechanisms underlying GABA-mediated stress responses and exploring potential GABA inhibitors as control measures.

## Data Availability

All data supporting the findings of this study are contained within the article; further inquiries can be directed to the corresponding author.

## Supplementary Materials

The following supporting information can be downloaded at: https://www.mdpi.com/article/10.3390/foods14020171/s1, Table S1: Primers used for construction of Δ*gabT* of *C. sakazakii*.
