# Supplementary material for: γ-Aminobutyric Acid (GABA) Metabolic Bypass Plays a Crucial Role in Stress Tolerance and Biofilm Formation in C. sakazakii ATCC 29544
Source: Foods. 2025 Jan 8;14(2):171. doi: 10.3390/foods14020171 (PMC11764851; doi:10.3390/foods14020171)
Supplement: Supplementary file 1 [file foods-14-00171-s001.zip › foods-3380799-supplementary.pdf]

Table S1 Primers used for construction of Δ*gabT* of *C. sakazakii*

Primers	Sequence (5'-3')
GabT-MF1	GGAATCTAGACCTTGAGTCGCTGCCGTTCGGGTTCACTTC
GabT-MF1	GCACCACTTTAAGGCAGGATGACTGCCACTGCTGATTGCTTTG
GabT-MF2	CAAAGCAATCAGCAGTGGCAGTCATCCTGCCTTAAAGTGGTGC
GabT-MR2	ACAGCTAGCGACGATATGTCAGCAGCACCACGAAAATCCA
GabT-TF	ATAGCCTCAATCAGCCCGTCG
GabT-TR	CGCAACACCGTAAATCACCAT
pLP-UF	GACACAGTTGTAACTGGTCCA
pLP-UR	CAGGAACACTTAACGGCTGAC
